# Complications of Coexisting Thymoma and Myelodysplastic Syndrome: A Case Report

**DOI:** 10.7759/cureus.70171

**Published:** 2024-09-25

**Authors:** Pamella Morello, Berta Martinez-Fortun, Mackenzie Fannin, Evan Sarmiento, Rodolfo Urruela, Sanika Chhabra, Silvia Sanchez Del Campo

**Affiliations:** 1 Osteopathic Medicine, Dr. Kiran C. Patel College of Osteopathic Medicine, Nova Southeastern University, Fort Lauderdale, USA; 2 Family Medicine, Larkin Community Hospital, Hialeah, USA; 3 Internal Medicine, Larkin Community Hospital, Hialeah, USA

**Keywords:** malignant thymoma, metastatic lung, multi-disciplinary care, myelodysplastic syndrome (mds), occupational risk exposure

## Abstract

A thymoma is a rare malignant tumor of the thymus gland, often associated with local invasion, recurrence, and autoimmune disorders. The interplay between thymoma and myelodysplastic syndrome (MDS) represents a complex clinical phenomenon, yet its underlying mechanisms and optimal management strategies remain incompletely understood.

We present the case of a 69-year-old male with recurrent thymoma, initially classified as type B1/AB, complicated by the subsequent development of MDS. The patient, who had worked as a plumber for over 20 years, had previously undergone robotic video-assisted thoracoscopic surgery (VATS), chemotherapy, immunotherapy, radiation, and stereotactic body radiation therapy (SBRT) in an attempt to treat the thymoma. Despite these interventions, the tumor recurred, invading the left lung pleura. Following his admission to our hospital, he experienced subsequent hospitalizations for anemia, recurrent pleural effusions, and leukocytosis, which were managed with blood transfusions, thoracentesis, and antibiotics. The onset of MDS in this patient raises questions about the potential interplay between thymoma and hematologic disorders, possibly related to immunological dysregulation, genetic predisposition, occupational exposure, or environmental factors.

This case illustrates the intricate link between recurrent thymoma and the onset of MDS. It emphasizes the necessity for further research to uncover their underlying mechanisms and identify novel therapeutic targets for this intriguing clinical entity. Additionally, the case reinforces the vital role of a multidisciplinary approach in treating both thymoma and MDS, as the complexities of care require collaborative efforts to ensure optimal patient outcomes.

## Introduction

Thymoma represents a malignant neoplasm primarily located in the mediastinum. Despite complete resection, it exhibits a propensity for recurrence. Numerous studies have explored the treatment modalities and prognostic indicators for recurrent thymoma, yet consensus remains elusive due to inadequate investigation into its risk factors. Notably, thymoma is associated with autoimmune disorders such as systemic lupus erythematous, Cushing's syndrome, syndrome of inappropriate antidiuretic hormone secretion (SIADH), and most commonly, myasthenia gravis (MG), with gene mutations implicated in its pathogenesis [1].

Thymomas are classified into subtypes based on their invasiveness, with non-invasive thymomas demonstrating a 10-year survival rate of 80%, compared to 30% for invasive variants. This case report describes a thymoma initially classified as low-risk. Despite this, the tumor exhibited aggressive progression and was further complicated by the onset of MDS, significantly complicating its management [2].

Thymoma, affecting fewer than five individuals per million, highlights the need for effective management strategies to guide future clinical practice and fill gaps in the medical literature. This article presents a case of recurrent thymoma in a male patient treated with a combination of surgical and nonsurgical approaches, alongside multimodal therapies, including chemotherapy. Notably, while chemotherapy is a recognized risk factor for hematological pathologies, the patient's occupational history is also considered a potential contributor to the development of MDS, underscoring the need for further research in this area.

## Case presentation

We present the case of a 69-year-old white Hispanic male with a medical history significant for basal cell carcinoma, major depressive disorder, hypertension, and chronic kidney disease. His family history includes the death of a son from sarcoma, though no genetic testing was performed on family members. Social history reveals that the patient was a lifelong non-smoker but with over 20 years of occupational exposure as a plumber. Notably, he worked without proper personal protective equipment, particularly in environments with polyvinyl chloride piping and associated dust.

The patient's initial presentation in the Fall of 2019 was prompted by significant dyspnea, leading to a chest x-ray revealing the sizable anterior mediastinal tumor exceeding 16 cm in dimension. Subsequent positron emission tomography (PET) and computed tomography (CT) imaging, as documented in the patient’s medical records, revealed a large lung mass with increased fluorodeoxyglucose (FDG) uptake. Although these PET/CT images are not available for inclusion due to external facility restrictions, the scan results were well-documented, showing increased FDG uptake along the anterior lateral aspect of the left lung pleura extending to the left peritoneum, suggestive of a suspected thymoma. The surgical intervention entailed left robotic video-assisted thoracoscopic surgery (VATS) for biopsy and tumor resection, resulting in permanent vocal cord paralysis due to the proximity of the tumor to the phrenic nerve. Pathological examination indicated a mixed lymphoid and epithelial neoplasm with positive immunohistochemical markers for PAX-8, p53, pankeratin, CD3, CD5, CD20, PAX-5, and Ki-67. The definitive diagnosis was established as thymoma type B1 or type AB, with considerations for subtype classification given the extensive mass size (13.1 cm). Postoperative treatment comprised external beam radiation therapy (EBRT) and chemotherapy; upon the availability of medical records, the specific chemotherapic drug administered was not available. Recurrence manifested in the Fall of 2021 as metastatic disease, necessitating a second mini-thoracotomy with biopsy, lung decortication, and bronchoscopy, mirroring initial pathology findings. In 2022, medical records confirmed increased FDG uptake along the anterolateral aspect of the left lung pleura, extending to the left peritoneum, indicating disease progression. Although PET/CT scans from that year are unavailable, the records noted that the tumor was deemed unresectable. The patient was treated with stereotactic body radiation therapy (SBRT) and chemotherapy, which he could not tolerate, leading to a switch to Nivolumab.

Per chart review, additional rounds of SBRT were administered until April 2023, when immunotherapy was halted due to thrombocytopenia, prompting a bone marrow biopsy revealing MDS. Treatment with Lenalidomide commenced in June 2023. The patient had an episode of neutropenia, leukopenia, and thrombocytopenia in August 2023 and was treated with Filgrastim. In September 2023, the patient was admitted to our hospital for chest tightness and dyspnea. The patient's medications included Lenalidomide 5 mg orally daily, and Nivolumab injections for the thymoma. On admission, vital signs were remarkable, with a pulse of 103 beats/min and O_2_ saturation of 93% in room air. Acute coronary syndrome workup was negative. Laboratory blood work is summarized in Table 1.

**Table 1:** Laboratory bloodwork results *Laboratory values outside of the reference range

	Laboratory Workup	Reference Range
*White blood count (WBC)	79600 cells/microL	4500-11000 cells/microL
*Neutrophils %	30%	40-60%
*Lymphocytes %	16.6%	20-40%
*Monocytes %	39.4%	2-8%
Basophils %	0.3%	0.3-1%
Eosinophils %	1.8%	1-4%
*Hemoglobin (Hgb)	5.8 g/dL	13.5-18.0 g/dL
*Mean corpuscular volume (MCV)	108 fL	80-100 fL
*Platelets	34000 mcL	150000-400000 mcL
*Absolute neutrophil count (ANC)	1277 neutrophils/microL	2500-7000 neutrophils/microL
Blood-urea-nitrogen (BUN)	20 mg/dL	7-20 mg/dL
Creatinine (Cr)	1.01 mg/dL	0.7-1.3 mg/dL
Alkaline phosphatase (ALP)	111 IU/L	44-147 IU/L
*Alanine aminotransferase (ALT)	73 U/L	4-36 U/L
*Aspartate aminotransferase (AST)	74 U/L	14-20 U/L
Estimated glomerular filtration rate (eGFR)	73 mL/min	>60 mL/min
*B-type natriuretic peptide (BNP)	7610 pg/mL	<100 pg/mL

**Figure 1:**
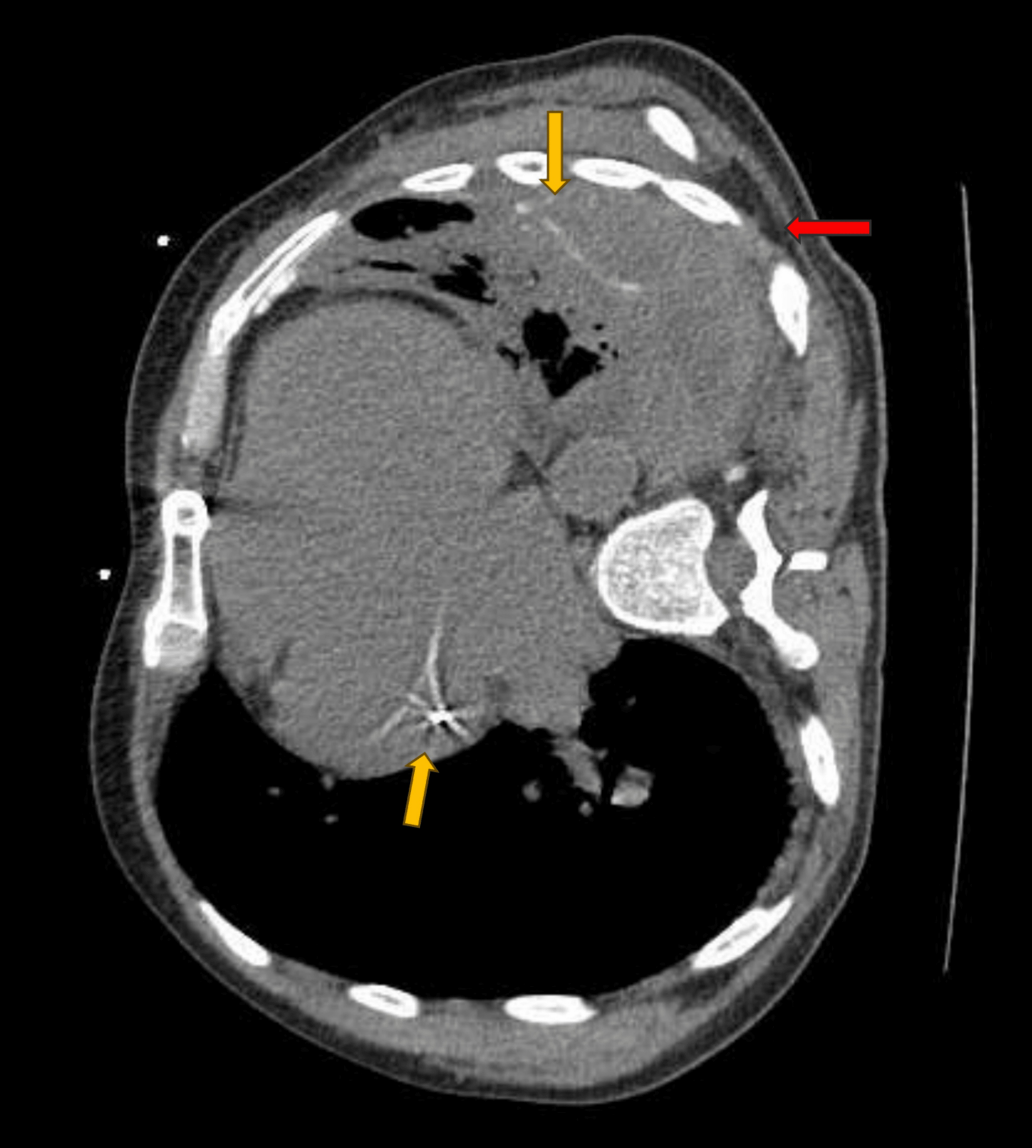
Cross-sectional view CT scan of chest without contrast Left-sided pleural thickening (red arrow) extending into the left minor fissure with irregularly shaped hyperdensities, which may represent amorphous calcifications (yellow arrows). Small-to-moderate right pleural effusion with tracking into the right major fissure. The left pleural findings are concerning for a neoplastic process.

Physical examination revealed diminished breath sounds bilaterally, with no rales, rhonchi, or wheezing. A chest X-ray showed pulmonary edema and a right-sided pleural effusion. Additionally, opacifications in the left lung, suggestive of malignancy, were noted in a patient with a known history of mediastinal cancer. A non-contrast CT scan of the chest revealed circumferential pleural thickening on the left side, most prominent in the posterior inferior region with extension into the left-sided fissure. These findings raised concerns for mesothelioma, although other neoplasms could not be completely excluded. Additionally, left lung consolidations were noted, likely due to infection or inflammation, with the possibility of an underlying neoplasm (Figure 1). 

**Figure 2:**
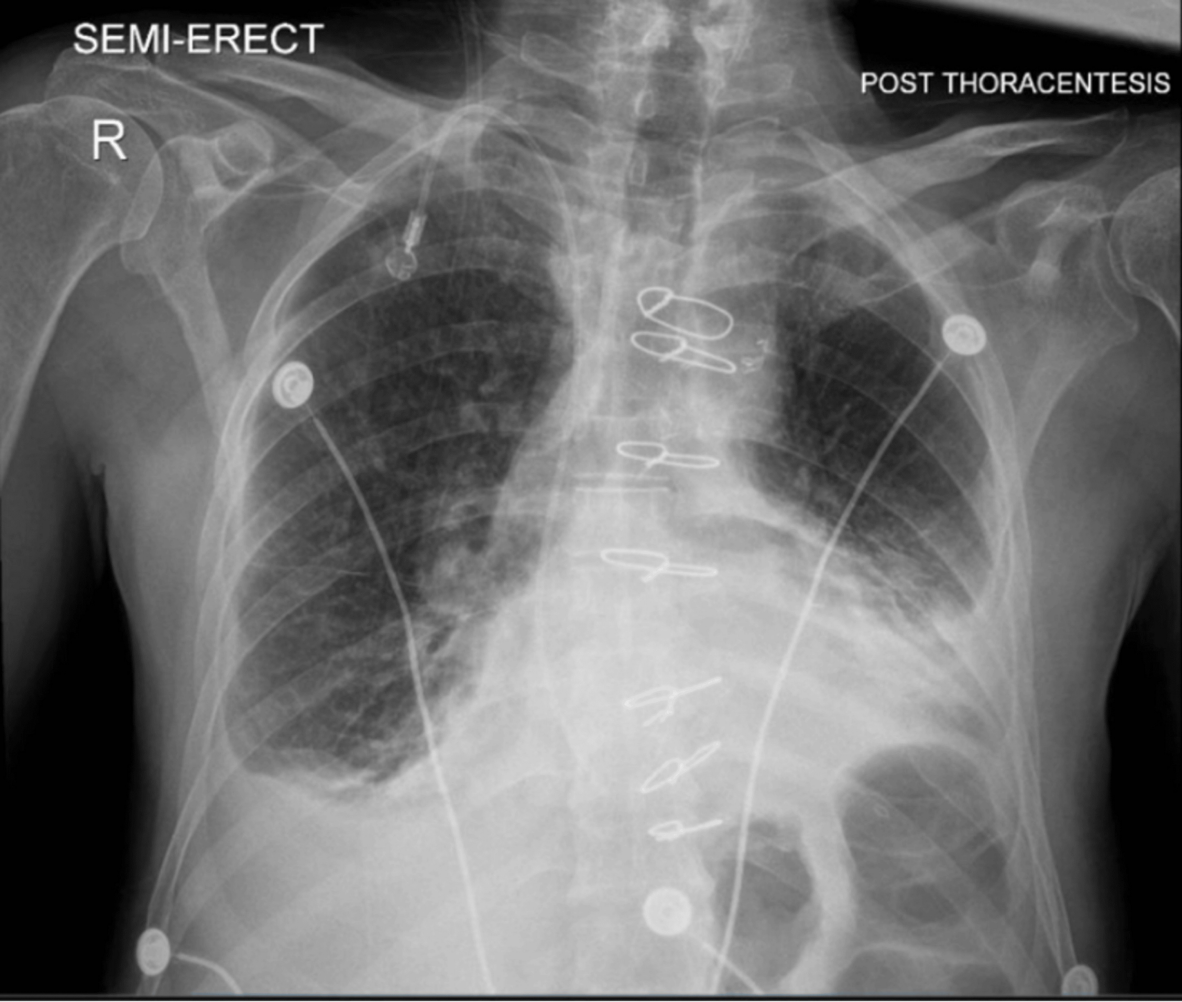
Post-thoracentesis single-view chest x-ray Lungs revealing reticular opacities. Ground-glass opacities throughout the right hemithorax. Presence of peripheral septal thickening and hilar vascular indistinctness. Pleura reveals obscuration over the right costophrenic angle and hemidiaphragm. Presence of hazy opacities overlying the left costophrenic angle and diaphragm. The cardio-mediastinal silhouette is enlarged. Mediastinal surgical clips. Seven intact median sternotomy wires. Findings are suggestive of pulmonary edema with small right-sided pleural effusion. No pneumothorax following thoracentesis.

Chest ultrasound (US) reinforced “moderate-to-large pleural effusion.” The patient was admitted for sepsis with acute hypoxemic respiratory failure, pleural effusion, and severe anemia. He was initially treated for pneumonia with Meropenem 1 gm IV Q8H and Levofloxacin 750 mg IV Q24H, along with nebulized Duoneb 3 mL INH RTQ8H and 1 unit of red blood cell transfusion. On his fifth day of hospitalization, the patient underwent ultrasound-guided right-side thoracentesis by the Interventional Radiology team. One liter of serosanguinous pleural fluid was extracted and sent for cytology analysis. The pleural fluid analysis was suggestive of a malignant process, consistent with metastatic involvement from the patient’s thymoma. The patient suffered no procedural complications and did note significant improvement in his dyspnea post-thoracentesis intervention (Figure 2). 

The patient was subsequently admitted for acute episodes of anemia and leukocytosis, requiring red blood cell transfusions and antibiotic treatment, respectively. Due to the tumor's unresectable nature and the hematologic complications, major surgery was ruled out, and further immunotherapy for the thymoma was delayed. Palliative care was initiated, focusing on symptom management through thoracentesis, pain control, and blood transfusions as needed. The patient continued his treatment with anti-depressants and was referred to outpatient psychiatry. This case underscores the significant challenges and complexities in managing recurrent thymoma alongside its associated comorbidities.

## Discussion

Thymoma and thymic carcinoma are rare manifestations of thymic epithelial tumors (TETs), arising from the outer surface of the thymus gland. Despite originating from similar cell types, they exhibit distinct clinical characteristics. Thymomas typically resemble normal thymic cells, displaying slow growth and infrequent metastasis. Conversely, thymic carcinoma is more aggressive, with cells exhibiting atypical morphology, increased metastatic potential, and greater therapeutic challenges [3]. The etiology of TETs remains largely unknown, although an association with multiple endocrine neoplasia type 1 (MEN1) has been documented. Thymoma is often associated with paraneoplastic syndromes, notably MG, whereas thymic carcinoma typically lacks these associations [4,5]. Additional paraneoplastic syndromes linked to thymoma include Good’s syndrome (thymoma-associated hypogammaglobulinemia) and autoimmune pure red cell aplasia, along with autoimmune disorders like Sjogren's disease and systemic lupus erythematosus [6].

Classification of thymoma tumors encompasses six distinct categories: Type A, characterized by spindle cell morphology; Type AB, comprising a mix of spindle cells and lymphocytes; Type B1, featuring a predominance of lymphocytes with scattered epithelial cells; Type B2, denoting cortical thymomas with a blend of lymphocytes and epithelial cells; Type B3, comprised mostly of epithelial cells arranged in sheets or nets; and Type C, representing thymic carcinoma with cytological atypia and invasive behavior. As the classification progresses from Type A to Type C, the tumor's aggressiveness escalates, reflecting a spectrum of malignancy within TETs [7]. Our case describes a patient previously diagnosed with mixed thymoma Type AB and B1 in 2019, without a history of paraneoplastic syndromes. Despite the initial pathology not indicating aggressiveness, the disease course was notable for two surgical interventions and multiple rounds of radiation and chemotherapy, yielding unsuccessful disease control. 

Furthermore, during medical treatment, the patient developed MDS with 5q deletion. MDS is associated with various factors including advanced age, environmental exposures (e.g., pesticides and benzene), genetic predisposition, bone marrow disorders, autoimmune diseases, viral infections (e.g., HIV and hepatitis), smoking, and exposure to ionizing radiation and chemotherapy [8]. The characteristic anemia with macrocytosis observed in our patient aligns with the 5q deletion in MDS [9]. While long-term chemotherapy and radiation exposure likely contributed to the diagnosis, exposure to vinyl chloride (due to occupational history as a plumber) may have also played a role, although its direct association with hematological disorders is not well-documented in the literature. Additionally, family history of cancer and the patient's prior history of basal cell carcinoma suggest a potential genetic predisposition.

Thymoma is often associated with hematological disorders like Good's syndrome and pure red cell aplasia [10-12]. Good's syndrome typically presents with leukopenia, which contrasts with our patient's findings, where thrombocytopenia and anemia, consistent with MDS, were observed. While the mechanisms linking thymoma and MDS with a 5q deletion remain unclear, immune dysregulation or genetic predisposition may contribute to their co-occurrence. In this case, no genetic testing was performed, and chemotherapy and immunotherapy used to treat the thymoma were suspected as the primary causes of the MDS development.

The management of thymoma and MDS 5q deletion may involve multimodal approaches, including surgery, radiation therapy, chemotherapy, immunosuppressive therapy, and supportive care measures. The optimal treatment strategy for patients with both conditions depends on various factors, including disease stage, extent of involvement, comorbidities, and individual patient preferences. However, further research is needed to elucidate the underlying mechanisms and potential causal relationship between thymoma and MDS and disease management.

The immune dysregulation inherent in patients with thymoma and hematological dysfunction predisposes these individuals to infectious diseases, as demonstrated in our patient. Pneumonia was identified on CT and chest x-ray and initially managed with Meropenem 1 gm intravenously every 24 hours. The medication regimen was later switched to Ertapenem 1 gm intravenously every 24 hours and Levofloxacin 750 mg intravenously every 24 hours under the guidance of the Infectious Disease team. Throughout the hospitalization, the white blood cell count trended down from 79600 cells/mL to 64000 cells/mL, while hemoglobin levels improved from 5.8 to 6.8 g/dL following the transfusion of 2 units of packed red blood cells. The patient remained on Lenalidomide 5 mg orally once daily for myelodysplastic syndrome (MDS) in addition to Ipratropium-Albuterol inhalation solution 0.5-2.5 3 mg/3 mL every six hours for respiratory improvement. The discharge occurred after a nine-day hospitalization, with arrangements made for home and healthcare assistance. Subsequently, the patient experienced two similar presentations necessitating readmission and corresponding treatment.

The comprehensive management of this patient involved a multidisciplinary team approach, including specialists from Infectious Disease, Cardiology, Pulmonology, Oncology, Hematology, and Primary Care. This approach necessitated extensive collaboration among medical teams to elucidate the patient's medical history and past therapeutic interventions. Effective communication among team members was essential in delivering optimal care.

## Conclusions

Overall, the coexistence of thymoma and MDS 5q deletion represents a complex clinical scenario requiring a multidisciplinary approach and personalized management strategies to address both neoplastic and hematologic manifestations. Close monitoring and long-term follow-up are essential to assess treatment responses, detect disease progression or recurrence, and optimize patient outcomes.
